# Melittin alleviates sepsis-induced acute kidney injury by promoting GPX4 expression to inhibit ferroptosis

**DOI:** 10.1080/13510002.2023.2290864

**Published:** 2023-12-27

**Authors:** Hongyan Zan, Jizheng Liu, Meixia Yang, Honghui Zhao, Chunyan Gao, Yunyan Dai, Zhiming Wang, Hongxuan Liu, Yunfei Zhang

**Affiliations:** a Departments of Emergency Internal Medicine, Shanxi Bethune Hospital, Shanxi Academy of Medical Sciences, Tongji Shanxi Hospital, Third Hospital of Shanxi Medical University, Taiyuan, People’s Republic of China; b Clinical laboratory, The Second Peoples Hospital of Liaocheng, Liaocheng, People’s Republic of China; c Clinical Laboratory, Shanxi Bethune Hospital, Shanxi Academy of Medical Sciences, Tongji Shanxi Hospital, Third Hospital of Shanxi Medical University, Taiyuan, People’s Republic of China; d Department of General Surgery, Shanxi Bethune Hospital, Taiyuan, People’s Republic of China

**Keywords:** Melittin, ferroptosis, GPX4, acute kidney injury, lipopolysaccharide

## Abstract

**Objectives:**

Melittin, the main component of bee venom, is a natural anti-inflammatory substance, in addition to its ability to fight cancer, antiviral, and useful in diabetes treatment. This study seeks to determine whether melittin can protect renal tissue from sepsis-induced damage by preventing ferroptosis and explore the protective mechanism.

**Methods:**

In this study, we investigated the specific protective mechanism of melittin against sepsis-induced renal injury by screening renal injury indicators and ferroptosis -related molecules and markers in animal and cellular models of sepsis.

**Results:**

Our results showed that treatment with melittin attenuated the pathological changes in mice with lipopolysaccharide-induced acute kidney injury. Additionally, we found that melittin attenuated ferroptosis in kidney tissue by enhancing GPX4 expression, which ultimately led to the reduction of kidney tissue injury. Furthermore, we observed that melittin enhanced NRF2 nuclear translocation, which consequently upregulated GPX4 expression. our findings suggest that melittin may be a potential therapeutic agent for the treatment of sepsis-associated acute kidney injury by inhibiting ferroptosis through the GPX4/NRF2 pathway.

**Conclusions:**

Our study reveals the protective mechanism of melittin in septic kidney injury and provides a new therapeutic direction for Sepsis-AKI.

## Introduction

Sepsis is a serious disease caused by an abnormal immune response to infection, which causes serious damage to the body's organs, including acute kidney injury (AKI). Acute kidney injury is a common complication of sepsis and a major cause of death in severely ill patients [1]. Renal tubular cell necrosis has been described as a histological feature and a major cause of acute kidney injury [2]. Lipopolysaccharide (LPS) is a kind of endotoxin secreted by gram-negative bacteria. Renal tubular epithelial cells are among the many cell types that are stimulated by this inflammatory mediator when it binds to cell-surface Toll-like receptors (TLRs), triggering oxidative stress, inflammation, and ultimately cell death [3].

Cell death is critical for normal development and internal environment homeostasis. It causes tissue damage, which can result in a variety of diseases, including acute kidney injury. Ferroptosis is a new type of regulating cell death named for the first time in 2012 by Brent R Stock well, which differs from the traditional morphological and biochemical reactions regulating cell death [4]. Reduction in cell viability, elevated intracellular iron and lipid peroxidation, and impaired antioxidant capability are all hallmarks of the cellular level process known as ferroptosis. In addition, ferroptosis has been linked to many physiological and pathological processes, such as cancer cell death, neurodegenerative diseases, AKI, drug-induced hepatotoxicity, and T-cell immunity [5]. It is vital to note that the primary pathological and physiological mechanisms of ferroptosis and necrosis in AKI involve renal tubular damage [6]. As a result, ferroptosis may represent a novel approach to the treatment of AKI.

Melittin, as a novel drug, plays a distinct role in the treatment of acute diseases [7]. In mice with endotoxin-induced acute kidney damage, melittin treatment reduced inflammation, oxidative stress, and cell death [8]. Furthermore, melittin has antioxidant and anti-inflammatory properties [9]. At the moment, the detailed mechanism of melittin on acute kidney injury caused by hypertoxicity is unknown. In this study, we hope to investigate melittin's inhibitory mechanism on ferroptosis and demonstrate that melittin is a promising drug for treating AKI.

## Materials and methods

### Cell lines

Human renal tubular epithelial cells (HK-2 cells) were obtained from service bio (Wuhan, China). The cells were maintained in 10% fetal bovine serum (C04001-500, Vivacell, China), 100 units/ml penicillin and 100μg/ml streptomycin DMEM/high glucose medium. The cells grew in a 37°C incubator with 5% CO_2_.

### Animals

Male wild-type (WT) mice aged 6–10 weeks (C57BL/6) were purchased from Animal Center of Shanxi Medical University (Shanxi, China) and raised in Experimental Center of Bethune Hospital of Shanxi Medical University. All procedures involving animals are approved by the Animal Policy and Welfare Committee of Shanxi Medical University.

### Model of LPS-induced AKI

Three groups of mice were randomly assigned: the wild-type injection group (WT), the LPS injection group (LPS), and the LPS plus melittin treatment group (LPS + Mel). LPS (10 mg/kg) was intraperitoneally delivered into the mice in the LPS group. The identical volume of ordinary saline was intraperitoneally delivered into the WT group of mice. One hour before the injection of LPS in the LPS + Mel group, melittin 0.01 mg/kg was administered intraperitoneally. All mice were executed at 24 h after drug administration. Quickly separate the kidneys, then puncture the heart to collect blood samples. For survival experiments, Mice were randomly assigned to one of three groups – Control, Lipopolysaccharide, or Lipopolysaccharide with melittin – each of which contained five mice. Melittin (0.01 mg/kg) was intraperitoneally given into mice an hour before the LPS (20 mg/kg) injection. The survival rate was tracked for 96 h following LPS treatment. Melittin was purchased from Seville (IM1270, Solarbio, China,), and lipopolysaccharide was obtained from Seville (IL2020, Solarbio, China)

### Biochemical analyses

For 10 min, plasma was separated from whole blood at 2500 Revolutions Per Minute (RPM). To assess renal function, the plasma concentrations of creatinine (RXWB0459-96, Ruixin Biotech, China) and blood urea nitrogen (BUN) (RXWB0153-96, Ruixin Biotech, China) were measured using the test kits.

### Lipid ROS assay and MDA assay

C11-BODIPY 581/591 (GC40165, Glpbio, USA) was used to measure the levels of lipid reactive oxygen species in a flow instrument. First, we stimulated the cells with different concentrations of LPS, LPS + Mel, Era (Erastin, An activator of ferroptosis) stimulants for 24 h, respectively, and then, 5-10 × 10^5^ cells were centrifuged at 2000rpm for 3 min at room temperature, and the supernatant was discarded. Each tube received 100 μl of C11-BODIPY probe at a final concentration of 2 μM and was incubated in a cell incubator for 30 min. Then, centrifuge at room temperature for 3 min at 2000rpm, discarding the supernatant. Each tube should be filled with 300 μl of PBS, centrifuged for 3 min at room temperature at 2000rpm, the supernatant discarded, and then two more times washed. 300 μl PBS was resuspended, stored in a 4°C refrigerator away from light, and detected in time by flow cytometry. Malondialdehyde (MDA) level was determined by MDA assay kit (BC0025, Solarbio, China)

### Histological analyses, immunofluorescence, and immunohistochemistry (IHC)

The kidneys were fixed in 4% paraformaldehyde and sent to Seville Company (Wuhan, China) for slices and stained with hematoxylin & eosin (HE) and Immunohistochemistry (IHC). Pathologists blindly examined the renal tubular injury and scored it based on the severity of the injury. 0 points equals 10% of tubules damaged; 1-point equals 11%−25% of tubules damaged; 2 points equals 26%−45% of tubules damaged; and 3 points equals 46%−75% of tubules damaged. Brush edge loss, cell vacuolation, cell desquamation, tubule dilatation, and tubule degeneration were all scored from 0 to 3, and the total tubule injury score was 15, with 15 being the highest value [10]. Obtain the image with Nikon microscope. Incubation was performed with the primary antibodies anti-NGAL antibody (PB9609, Boster, China) and anti-4-HNE antibody (AO2685-4, Boster, China). Use Case Viewer software (Sysmex Products, Europe) to view.

### Immunofluorescence assay

After being stimulated with 10μg/μl of LPS and 1μg/μl of melittin, the cells were fixed with 4% formaldehyde. After fixation, they were incubated with an anti-NRF2 antibody (ab92946, abcam, USA) at a dilution of 1:400 for 1 h. Following this, the cells were incubated with AF488 sheep anti-rabbit antibody at 4° for 30 min. DAPI staining was performed for 5 min before observation using a Nikon inverted fluorescence microscope.

### RT–PCR

RNA was extracted from cells or tissues using the Rapid RNA Extraction Kit (SM129-02, Sevenbio, China), and cDNA was created through reverse transcription using the cDNA Kit (SM134-01 (50 T), Sevenbio, China). The Bio-Rad CFX96 was used for quantitative PCR, and transcription levels were quantified using a cycle threshold (ct). The quantitative index is 2^−ΔΔct^.

**Table:** 

Primer	Sequence 5′−3′	BLAST RID
*GPX4* RT-qPCR(F)	GAGGCAAGACCGAAGTAAACTAC	KVGU74F801N
*GPX4* RT-qPCR(R)	CCGAACTGGTTACACGGGAA	KVGU74F801N
*NRF2* RT-qPCR(F)	AGAGGACCTGGTGAGGGATAC	KVXM2SUT013
*NRF2* RT-qPCR(R)	CTTCCAGAAGGCATGTTGAC	KVXM2SUT013
*GAPDH* RT-qPCR(F)	GCAAATTCCATGGCACCGT	KVYF4KGA016
*GAPDH* RT-qPCR(R)	GCCCCACTTGATTTTGGAGG	KVYF4KGA016
*SLC7A11* RT-qPCR(F)	TGCTGGGCTGATTTTATCTTCG	KVZ2C5EE01N
*SLC7A11* RT-qPCR(R)	GAAAGGGCAACCATGAAGAGG	KVZ2C5EE01N
*Gpx4* RT-qPCR(F)	TGTGCATCCCGCGATGATT	KW00WZH3013
*Gpx4* RT-qPCR(R)	CCCTGTACTTATCCAGGCAGA	KW00WZH3013
*Nrf2* RT-qPCR(F)	AAGCACAGCCAGCACATTCTCC	KW0GWZJ0013
*Nrf2* RT-qPCR(R)	TGACCAGGACTCACGGGAACTT	KW0GWZJ0013
*Gapdh* RT-qPCR(F)	GGTGAAGGTCGGTGTGAACG	KW117WD4016
*Gapdh* RT-qPCR(R)	CTCGCTCCTGGAAGATGGTG	KW117WD4016
*Slc7a11* RT-qPCR(F)	TCCTGCTTTGGCTCCATGAACG	KW1SRCRF016
*Slc7a11* RT-qPCR(R)	AGAGGAGTGTGCTTGCGGACAT	KW1SRCRF016

### Western blotting

The gel was prepared according to the SDS-PAGE gel fast preparation kit instructions. The electrophoresis conditions were set to constant voltage 90 V for 30 min and 180 V for 55 min until the sample ran to the membrane transfer. After the transfer, the NC membrane was rinsed briefly in TBST and blocked in blocking solution at room temperature for 1 h. The membrane was then cut into one target protein band and washed with TBST. The specific protein primary antibody was diluted with commercial primary antibody dilution solution and added to the corresponding protein band. The band was incubated overnight at 4°C and then washed three times with TBST for 5 min each. The secondary antibody was diluted with TBST containing 5% skimmed milk and added to the target protein band after washing. The band was incubated at room temperature for 1 h and then washed three times with TBST for 10 min each. The membrane was washed with TBST after incubating with the secondary antibody and then developed and analyzed. The primary antibodies utilized in this study were anti-NRF2 antibody (ab92946, abcam, USA) and anti-GPX4 antibody (52455S, Cell Signaling Technology, USA). Goat Anti-Rabbit IgG (H + L) was employed as a secondary antibody (SA00001-2, proteintech, China).

### TdT-mediated dUTP nick end labeling (TUNEL) assay

Apoptosis of renal tubular cells in kidney slices was identified by TUNEL staining using a one-step cell death detection kit according to the instructions of the CF488 TUNEL Cell Apoptosis Detection Kit (G1504-50 T, Sevenbio, China). Tissue sections were dewaxed, proteinase K treated and cleaned. Then, the slices were detected with TUNEL reaction mixture. Cells stained with TUNEL were counted in 10 arbitrarily selected non-overlapping (400 times magnification) of each kidney.

### Detection of cell nuclear proteins

The cells (5 × 10^6^∼1 × 10^7^) were washed twice with precooled PBS and lysed with cytoplasmic protein extraction buffer on ice using ultrasound and vortexing for 10 s. After incubating on ice for 20 min with intermittent vortexing (3–5 times), the lysates were centrifuged at 4°C, 12,000rpm for 10 min and the supernatants were collected as the cytoplasmic protein fractions. To prevent nuclear protein contamination, this process was repeated twice with cytoplasmic protein extraction buffer. The nuclear proteins were extracted with precooled nuclear protein extraction buffer on ice using vortexing for 10 s and incubating on ice for 10 min with intermittent vortexing (2–3 times). The nuclear extracts were centrifuged at 4°C, 12,000rpm for 10 min and the supernatants were collected as the nuclear protein fractions. The fractions were aliquoted and stored at −80°C to avoid repeated freezing and thawing.

### Detection of Fe2+

The cell samples (5 × 10^6^) were washed twice with precooled PBS and lysed with 100–200 μL of lysis buffer per well using an ultrasonic disruptor. The lysates were centrifuged at 8000 g for 10 min and the supernatants were assayed. The samples and standards were mixed in different groups according to the manufacturer's instructions, incubated with the detection solution at room temperature for 20 min, and the absorbance was measured at 510 nm.

### Statistical analysis

All data followed a normal distribution. Statistical significance between groups was determined using one-way ANOVA (Prism 9.0. 0, GraphPad Software). The statistical significance was set as *P* < 0.05. Gray quantification of Western blot stripes was performed using Image J software to compare protein expression levels.

## Results

### Melittin attenuates LPS-induced renal dysfunction

**Figure 1.**
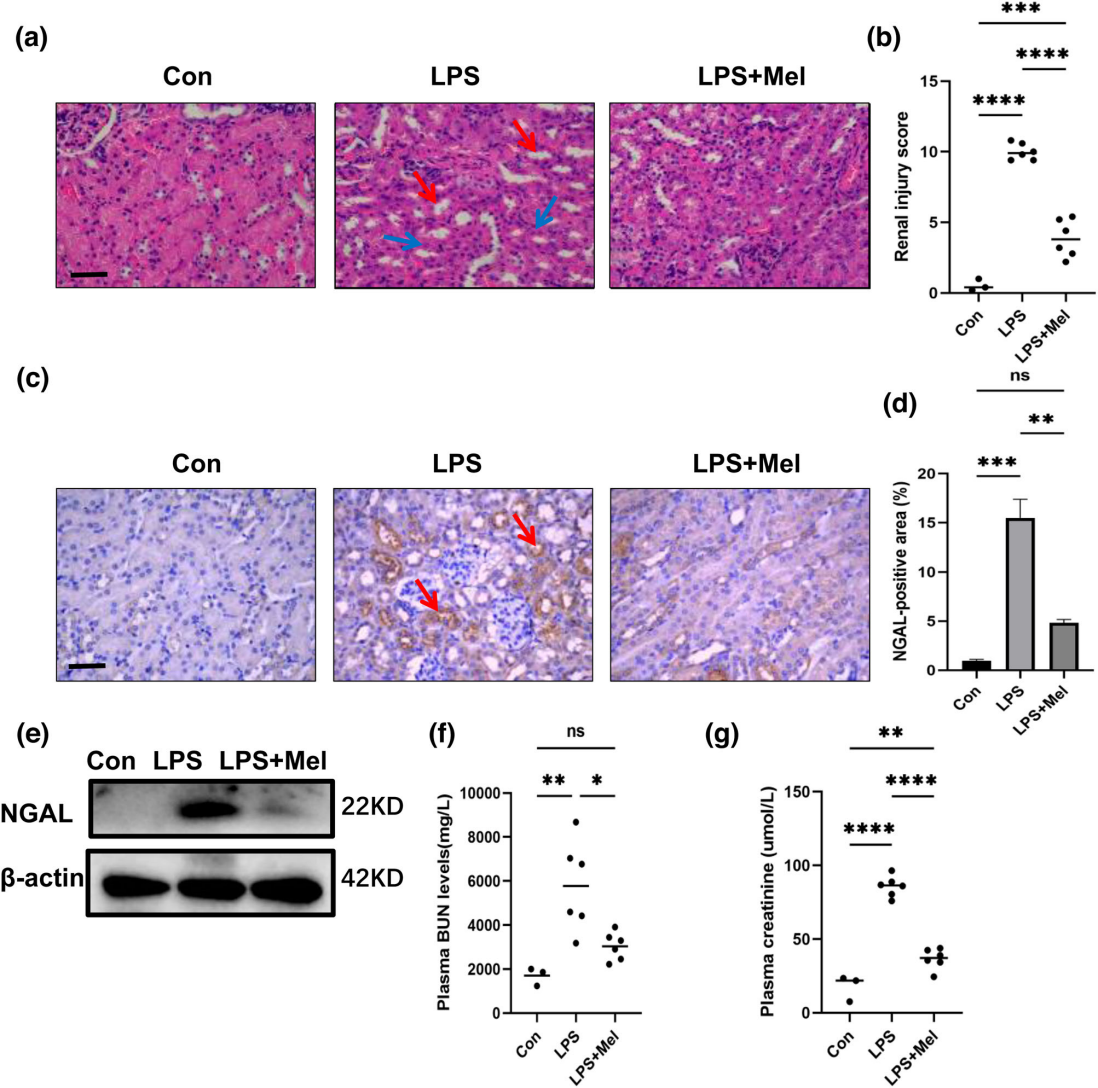
Effect of Melittin Attenuates sepsis-AKI. (a) H&E staining of kidney tissues from the Different groups. The red arrows denote the occurrence of tubular dilatation. The blue arrows highlight the swelling of renal tubular epithelial cells. Scale bars: 400 μm. (b) Tubular injury score. (c) Immunohistochemical staining of kidney with primary antibodies against NGAL. The areas marked with red arrows are NGAL-positive regions. Scale bars: 400 μm. (d) Average percentage of positive staining for NGAL per field.(n = 3). (e) Western blotting of NGAL in kidney tissues. (f) Plasma BUN levels. (g) Plasma creatinine levels. *P < 0.05, **P < 0.01, ***P < 0.001, ****P < 0.0001; ns, no significant difference between control and LPS group.

To track renal function, serum creatinine and urea nitrogen levels were assessed 24 h after LPS injection. Lipopolysaccharide-treated animals exhibited an increase in hemorrhagic creatinine and BUN concentration after receiving LPS intraperitoneally for 24 h. Two of the most common biochemical indicators used in clinical diagnostics to assess acute renal injury are serum creatinine and blood urea nitrogen levels [11]. The indications, however, demonstrated a declining trend in -melittin-injected mice (Figure 1(f) and (g)). Renal tubular epithelial cell edema, acute tubular dilatation, and substantial tubular development were all seen in LPS-treated animals but not in the melittin-treated group, as shown by H&E staining (Figure 1(a)). These findings are consistent with those of the tubular damage index, which was raised after LPS administration and reduced after melittin injection.

To go further into the function of melittin in kidney damage, we looked into the protein expression of neutrophil gelatinase-associated lipid transport protein (NGAL), a tubular injury marker of early kidney injury. Western blotting demonstrated that melittin successfully suppressed the expression of indicators of renal tubular damage, and it also showed that melittin effectively controlled the increased NGAL protein in LPS-injected animals. These finding demonstrates that melittin may reduce sepsis-related acute renal damage.

### Melittin protects against sepsis-AKI through attenuating ferroptosis

**Figure 2.**
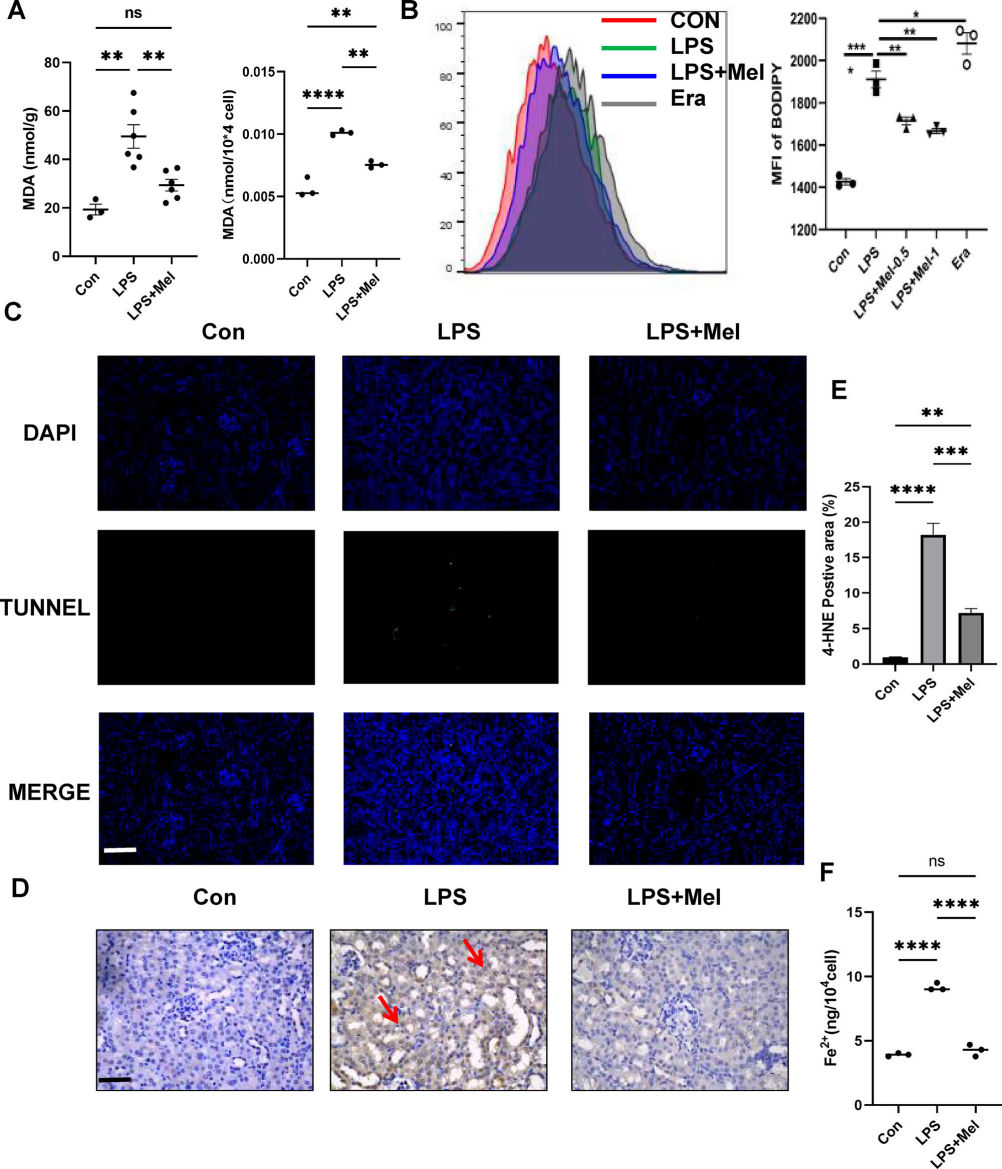
Melittin protects Against sepsis-AKI through Attenuating Ferroptosis. (a) MDA levels in animal model and HK-2 cells. (b) HK-2 cells were treated with drugs for 24 h and lipid ROS level was assayed by flow cytometry (n = 3). (c) TUNEL staining of kidney tissues from three group. Scale bars: 400 μm. (d) Immunohistochemical staining of kidney with primary antibodies against 4-HNE. The areas marked with red arrows are 4-HNE-positive regions. Scale bars: 400 μm. (e) Average percentage of positive staining for 4-HNE per field (n = 3). (f) Expression level of Fe^2+^ in HK-2 cells.*P < 0.05, **P < 0.01, ***P < 0.001, ****P < 0.0001; ns, no significant difference between control and LPS group.

Ferroptosis is a distinct form of regulated cell death that is closely associated with various diseases [12]. One of the hallmarks of ferroptosis is lipid oxidation, and therefore we investigated the lipid-related products and reactive oxygen species in cells. In LPS-treated HK-2 cells, melittin effectively reduced Malondialdehyde (MDA), one of the end products of lipid peroxidation, which was consistent with animal model verification (Figure 2(a)). Treatment of HK-2 cells with ferroptosis inducer-Erastin (Era) 2μmol/l resulted in the increase of cell ROS, while melittin treatment stopped the increase of lipid ROS. 4-hydroxynonena (4-HNE) is also a common end product of lipid peroxidation and is used as a reliable indicator in lipid peroxidation assays [13]. Immunohistochemical staining of kidney sections with anti-4-HNE antibody showed results that were consistent with other lipid peroxidation indicators. However, only the cortical tubular epithelium in the renal tissue showed an increased 4-HNE-positive area, and the glomeruli appeared unaffected (Figure 2(d)). Furthermore, iron accumulation is another hallmark of ferroptosis. In the group treated with Melittin, the increase in iron content caused by LPS was effectively inhibited by Melittin (Figure 2(f)). Apoptosis plays a crucial role in the pathophysiology of lipopolysaccharide-induced kidney injury, and we investigated the effect of melittin on lipopolysaccharide-induced apoptosis and cell death. The results showed a significant increase in the number of TUNEL-stained cells in the kidneys of mice injected with LPS. However, melittin significantly reduced the number of TUNEL-stained cells (Figure 2(c)). Thus, we hypothesize that melittin may ultimately alleviate LPS-induced acute kidney injury by reducing ferroptosis and apoptosis and cell death in kidney tissue.

### Melittin promotes Gpx4 expression to attenuate ferroptosis in sepsis-AKI

**Figure 3.**
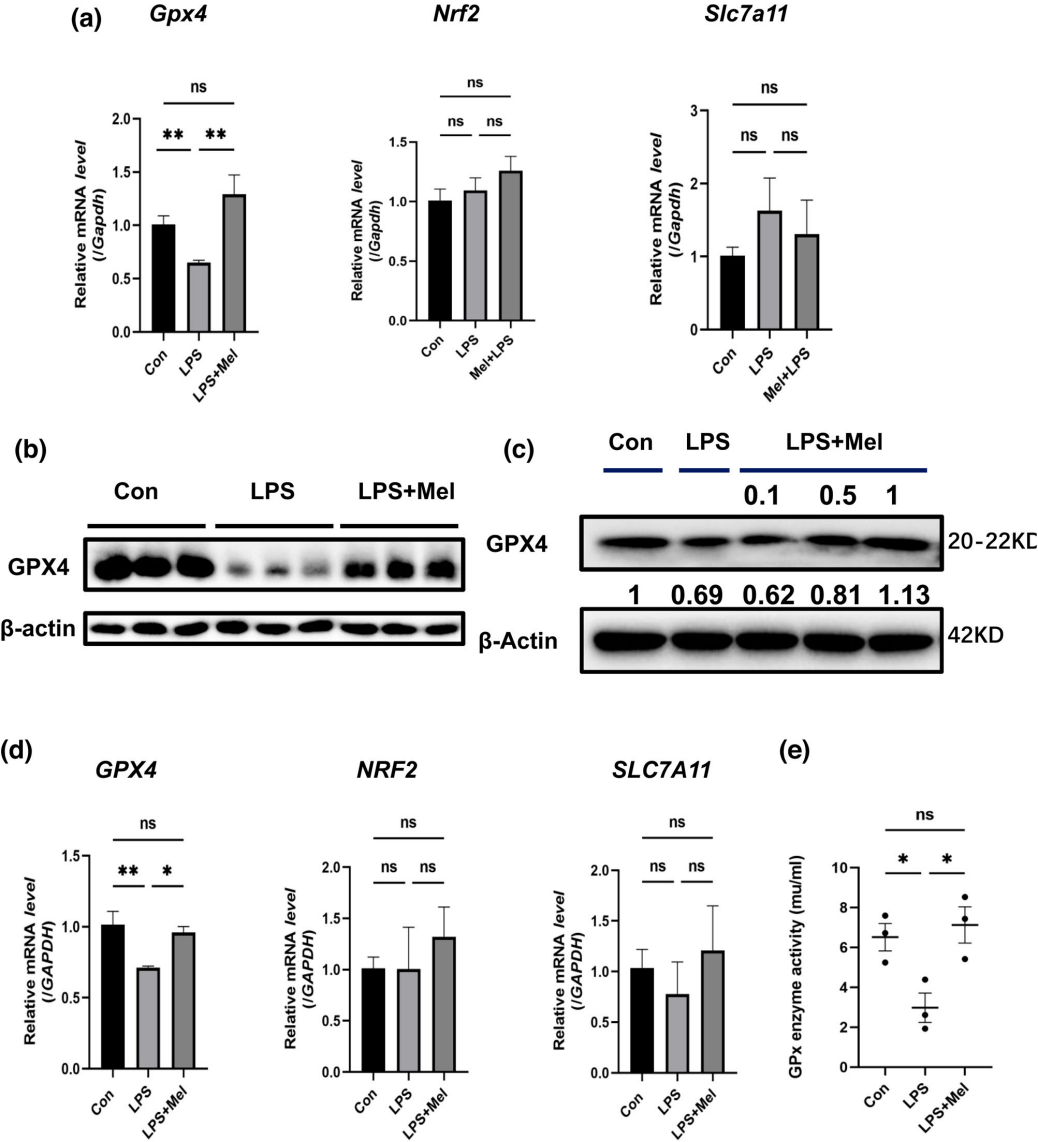
Melittin promotes Gpx4 expression to Attenuate Ferroptosis in sepsis-AKI. (a) Expression levels of *Gpx4, Nrf2* and *Slc7a11* in animal model determined by RT-qPCR. In expression levels of *Gpx4:WT* group:n = 3, LPS and LPS + Mel group:n = 5*,* the expression levels of *Nrf2* and *Slc7a11:*n = 3 per each group. (b, c) Expression of GPX4 in animal and HK-2 cells by western blotting. The values reported for the LPS group and LPS + Mel group were ratios of gray values in comparison to the control group. (d) Expression levels of *GPX4, NRF2* and *SLC7A11* in HK-2 Cells examined by RT-qPCR. Expression levels of *GPX4:n = 5* per each group*,* expression levels of *NRF2* and *SLC7A11:n = 3* per each group. (e) Gpx Enzyme activity (n = 3). *P < 0.05, **P < 0.01; ns, no significant difference between control and LPS group.

To investigate the molecular mechanism by which melittin attenuates sepsis-AKI by inhibiting ferroptosis, we examined the function of the cystine/glutamate transporter system since ferroptosis depends on its proper functioning. We first evaluated the effect of system Xc^-^ specific subunit *SLC7A11* and found that there was no significant difference in *SLC7A11* levels among the groups (Figure 3(a, d)). Next, we investigated the antioxidant capacity of cells, and GPX4, a critical component of the antioxidant defense, is also an essential regulator of ferroptosis [14]. In addition, NRF2 has been shown to reduce ferroptosis in HCC by enhancing antioxidant proteins (such as quinone oxidoreductase 1 and heme oxygenase-1 (HO-1)) in cells [15]. As indicated in Figure 3(a, d), melittin did not affect the level of *NRF2* in both animal and cellular models. However, the levels of GPX4 and its mRNA were significantly decreased in HK-2 cells and animal models in the LPS group compared to the control group. After melittin treatment, *GPX4* levels were increased in both HK-2 cells and mouse kidney tissues. We measured the activity of the Gpx enzyme in three experimental groups. The results indicate that Melittin enhanced the activity of the GPX enzyme, countering the toxic effects of LPS (Figure 3(e)). Therefore, we suggest that melittin attenuates ferroptosis in kidney injury by promoting GPX4 expression.

### Melittin promotes Gpx4 expression through enhancement of nuclear transformation of NRF2

**Figure 4.**
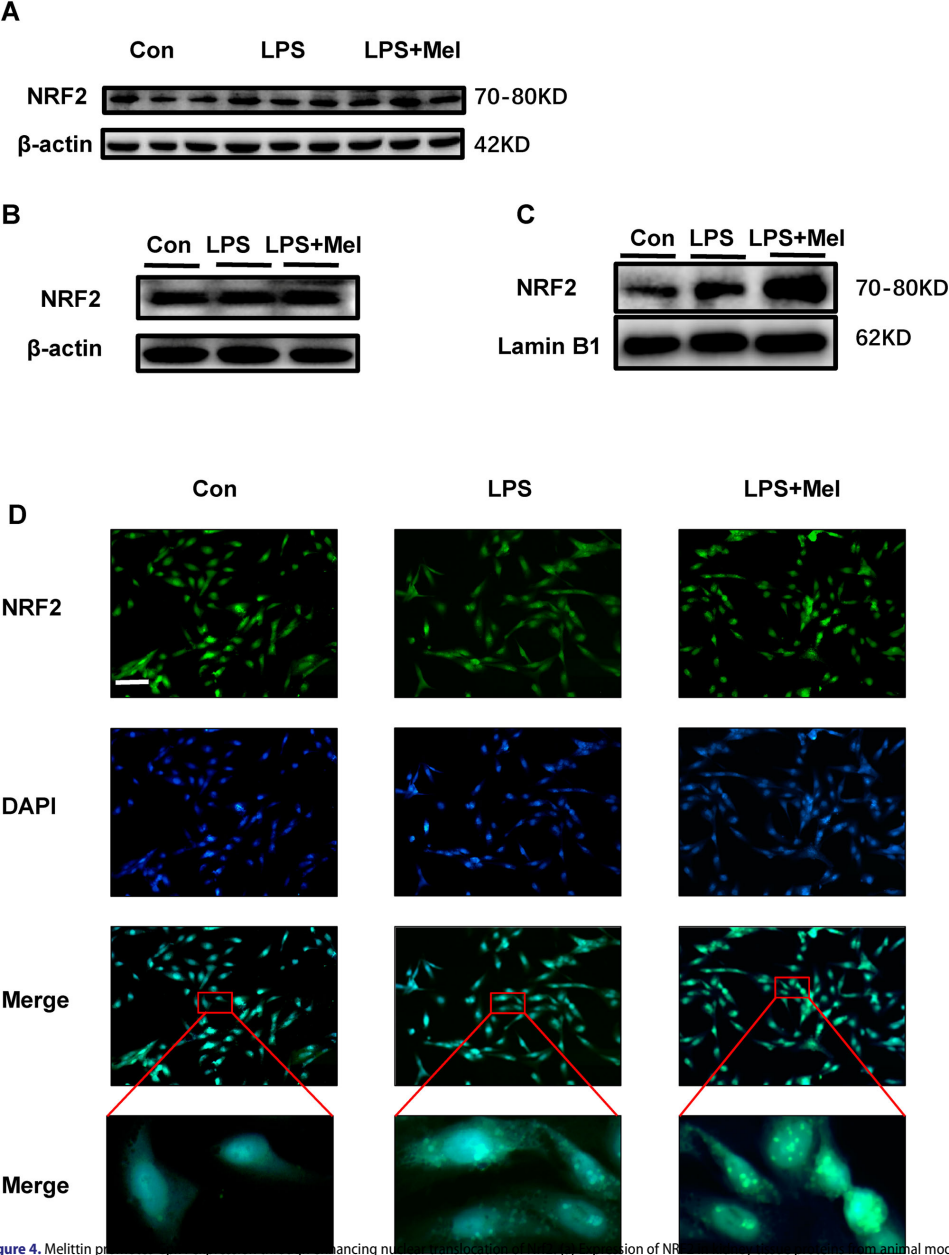
Melittin promotes Gpx4 expression through enhancing nuclear translocation of Nrf2. (a) Expression of NRF2 in kidney tissue proteins from animal models (n = 3). (b) Expression of NRF2 in the total protein of HK-2 cells. (c) Expression of NRF2 in nuclear proteins of HK-2 cells. (d) Representative immunofluorescence images of Nrf2 in HK-2 cells.

It has been reported that NRF2 is an essential transcription factor responsible for the maintenance of cellular metabolism, redox, and iron metabolism proteins, particularly in catalyzing glutamate-cysteine ligases, require the involvement of NRF2 [16]. Additionally, GPX4 is one of the downstream targets of NRF2 [16]. In cellular and animal experiments, there was no significant difference in Nrf2 protein expression in the LPS plus Mel group (Figure 4(a, b)). Therefore, we speculated that NRF2 may enter the nucleus to initiate antioxidant function. Subsequently, we determined the level and location of NRF2 in the LPS plus Mel group through fluorescence microscopy. The results in Figure 4(d) display that after LPS stimulation, NRF2 partially enters the nucleus, whereas after melittin stimulation, NRF2 is expressed at high levels in the nucleus. We extracted the nucleus protein after melittin stimulation and found that the level of NRF2 was significantly elevated, implying that melittin may be dependent upon the upregulation of NRF2 for promoting GPX4 expression in sepsis-AKI.

### Melittin impact survivor in sepsis-induced sepsis model

**Figure 5.**
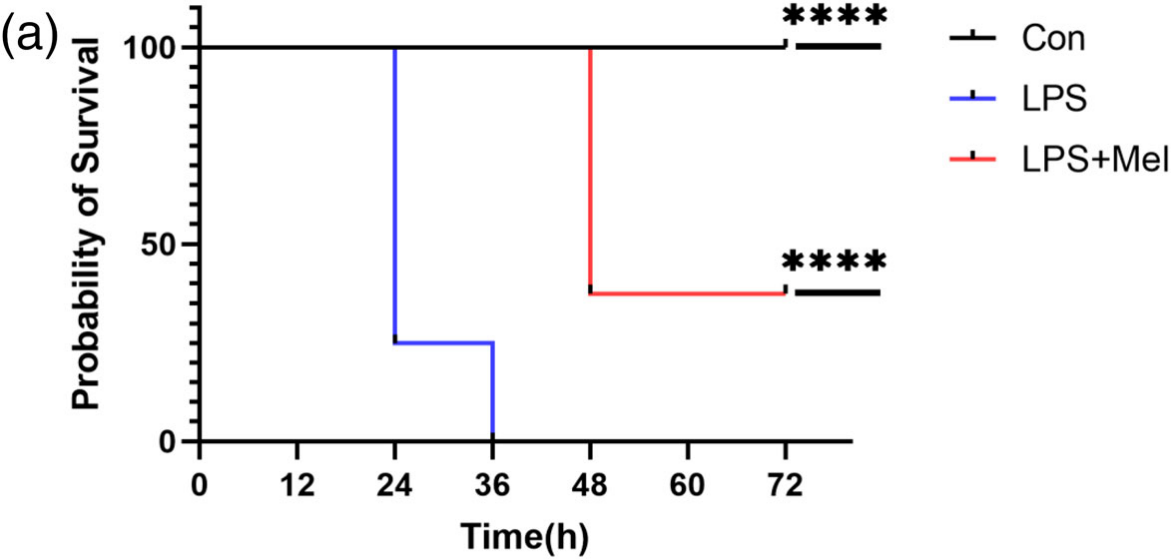
Melittin improves survival in sepsis-induced sepsis Model. (a) Melittin intraperitoneally administered at a dose of 0.01 mg/kg one hour prior to injecting lipopolysaccharide (LPS) at a dose of 20 mg/kg. Mice in the control group received an intraperitoneal injection of vehicle (saline). n = 8 per each group. **P < 0.01 compared with the LPS-injected group (LPS).

Finally, mice were administered melittin intraperitoneally at a dose of 0.01 mg/kg before and after receiving a 20 mg/kg LPS injection in order to assess the survival rate of LPS-injected mice (Figure 5(a)). The findings demonstrated that melittin therapy greatly increased mice's survival rates.

## Discussion

The high mortality rate among severely ill patients has traditionally been significantly attributed to sepsis. The poor prognosis of acute and severe patients is also directly associated with sepsis-AKI [17]. However, there is no effective treatment for sepsis-induced kidney damage. A novel medication called melittin has long been used to treat both acute and chronic illnesses. Researchers discovered that melittin operates as an antibiotic, has an antibacterial effect, and also helps people from getting sick from viruses by decreasing their infectivity [18]. Previous studies have shown that melittin can ameliorate the acute renal damage that lipopolysaccharide induces in mice [8]. In conclusion, melittin is a recently developed medication that shows promise as a Sepsis-AKI treatment.

Cell death is a fundamental aspect of regulating cell life and is crucial for maintaining the normal functioning of the organism [19]. Ferroptosis is a recently discovered mode of cell death, involving iron-dependent membrane lipid damage and many metabolic pathways controlling the sensitivity of cells to ferroptosis, including iron, lipids, and amino acids [20]. In this study, we demonstrated that melittin inhibits the decline in renal function, as evidenced by reduced levels of plasma creatinine and urea nitrogen, and structural damage to renal tissues, such as tubular dilatation and tubular cell swelling. Additionally, the expression of NGAL, a marker of renal tubular injury, was significantly reduced. Furthermore, we found that melittin attenuated the effect of Era on lipid oxidation in HK-2 cells, similarly to Fer-1, an ferroptosis inhibitor. Therefore, we postulate that melittin attenuates Sepsis-AKI by inhibiting ferroptosis.

**Figure 6.**
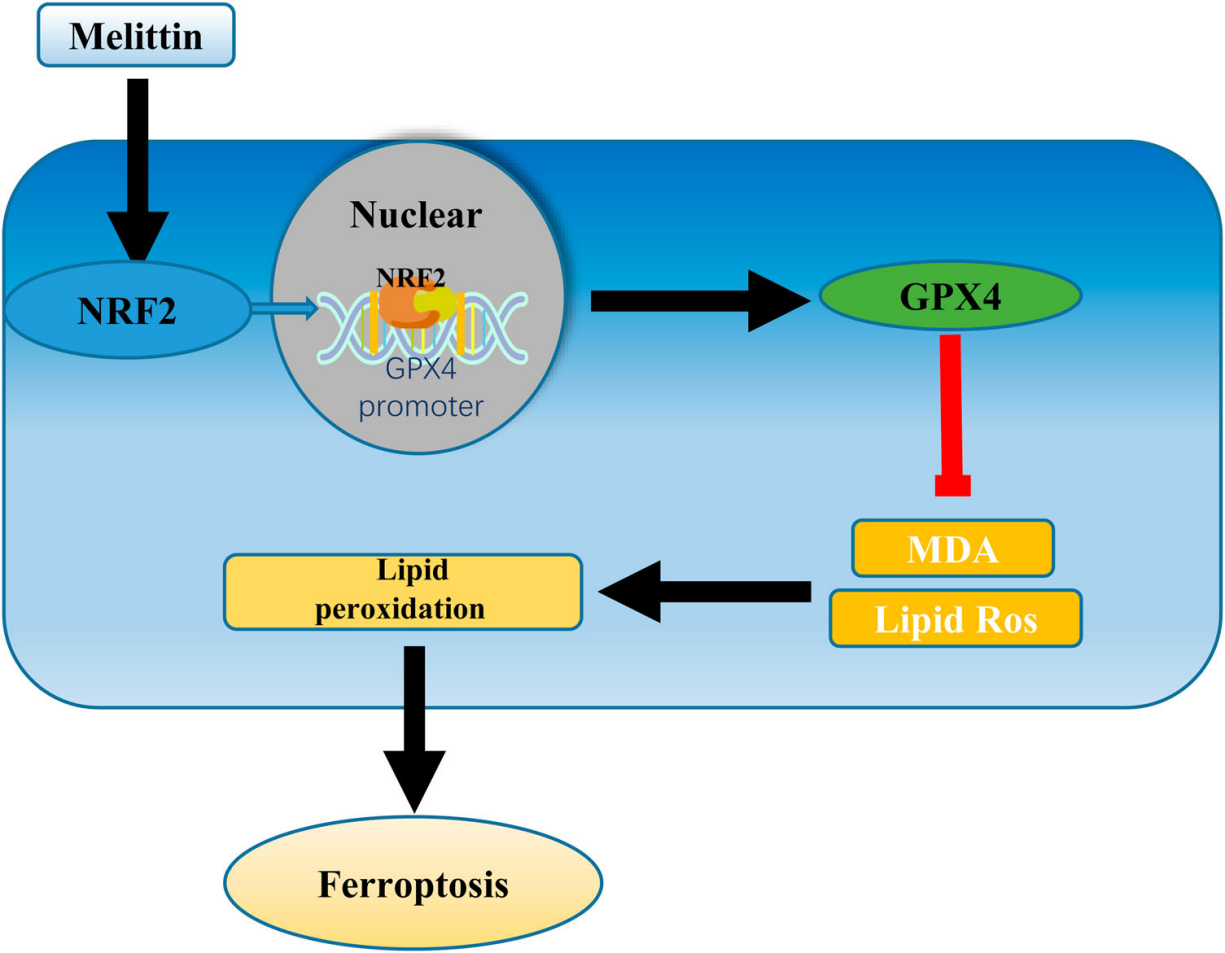
Graphical abstract. A model of renal injury was created by inhibiting lipopolysaccharide with melittin. Melittin promotes the expression of GPX4 by facilitating the nuclear translocation of NRF2, enhancing antioxidant capacity, reducing ferroptosis, and ultimately mitigating sepsis-induced acute kidney injury.

Gpx4, which resists lipid peroxidation during ferroptosis, was the first central inhibitor found to inhibit ferroptosis [21]. Since Gpx4 is a selenoprotein, selenium may also play a role in the inhibition of ferroptosis, as demonstrated previously [22]. Since Gpx4 plays an important role in resisting lipid peroxidation, we verified the reduced Gpx4 expression by Western blotting and RT–PCR in HK-2 cells and LPS-induced sepsis-AKI model mice, respectively. It has been reported that Nrf2 can target and regulate Gpx4, and among the tumor suppressors that regulate ferroptosis, Nrf2 regulates the level of SLC7A11 to control the expression of GPX4 [23]. Normally, Nrf2 binds to KEAP1, but in the presence of oxidative stress, Nrf2 dissociates from the KEAP1 protein and enters the nucleus to express antioxidant [23]. Nrf2 activates the antioxidant response element (ARE) and increases the transcription of Nrf2-regulated genes such as Heme oxygenase-1 (HO-1), Gluthathione S-tranferase (GST), and Superoxide dismutase (SOD). HO-1 can cooperate with NADPH cytochrome P450 to degrade heme to produce iron ions, carbon monoxide and biliverdin, which are antioxidants that can protect tissues from oxidation. SOD can catalyze the dismutation of superoxide radicals (O^2-^) into molecular oxygen (O_2_) or hydrogen peroxide (H_2_O_2_), thus exerting an antioxidant effect. Among the cell protective genes regulated by Nrf2 transcription factor, Gluthahione peroxidase plays an important role. We observed an increase in the nuclear expression level of NRF2 using a fluorescence microscope [24]. Therefore, we suggest that NRF2 up regulates GPX4 expression levels to attenuate AKI caused by LPS. According to our research findings, the mechanism by which Melittin regulates GPX4 through the activation of Nrf2 may be illustrated as shown in the Figure 6.

## Conclusions

Our findings demonstrate that melittin, particularly in the inflammatory response, oxidative stimulation, and lipid peroxidation, can reduce ferroptosis and withstand acute kidney damage and mortality brought on by endotoxin. This is fresh proof that melittin may be utilized to stop sepsis-related consequences.

## Data Availability

The data used to support the findings of this study are avail-able from the corresponding author upon request.
